# Isoliquiritigenin Confers Neuroprotection and Alleviates Amyloid-β42-Induced Neuroinflammation in Microglia by Regulating the Nrf2/NF-κB Signaling

**DOI:** 10.3389/fnins.2021.638772

**Published:** 2021-02-11

**Authors:** Yue Fu, Jianping Jia

**Affiliations:** ^1^Innovation Center for Neurological Disorders and Department of Neurology, Xuanwu Hospital, National Clinical Research Center for Geriatric Diseases, Capital Medical University, Beijing, China; ^2^Beijing Key Laboratory of Geriatric Cognitive Disorders, Beijing, China; ^3^Clinical Center for Neurodegenerative Disease and Memory Impairment, Capital Medical University, Beijing, China; ^4^Center of Alzheimer’s Disease, Beijing Institute of Brain Disorders, Collaborative Innovation Center for Brain Disorders, Capital Medical University, Beijing, China; ^5^Key Laboratory of Neurodegenerative Diseases, Ministry of Education, Beijing, China

**Keywords:** Alzheimer’s disease, Amyloid-β oligomers (AβOs), inflammation, oxidative stress, Isoliquiritigenin

## Abstract

**Background:**

Neuroinflammation and oxidative stress are two major pathological characteristics of Alzheimer’s disease (AD). Amyloid-β oligomers (AβO), a toxic form of Aβ, promote the neuroinflammation and oxidative stress in the development of AD. Isoliquiritigenin (ISL), a natural flavonoid isolated from the root of liquorice, has been shown to exert inhibitory effects on inflammatory response and oxidative stress.

**Objectives:**

The main purpose of this study is to assess the influence of ISL on inflammatory response and oxidative stress in BV2 cells stimulated with AβO, and to explore the underlying molecular mechanisms.

**Methods:**

3-(4,5-dimethyl-2-thiazolyl)-2, 5-diphenyl-2-H- tetrazolium bromide (MTT) and lactate dehydrogenase (LDH) cytotoxicity assays were used to assess the toxic or protective effects of ISL. The expression levels of interleukin-1β, interleukin-6, and tumor necrosis factor-α were assessed by quantitative real-time polymerase chain reaction (qRT-PCR) and enzyme-linked immunosorbent assays. Morphological changes in BV2 cells were assessed by immunofluorescence method. Nitric oxide (NO) assay kit was used to determinate the NO production. Western blot, qRT-PCR and immunofluorescence were used to explore the underlying molecular mechanisms.

**Results:**

ISL treatment reduced the production of inflammatory cytokines and NO, and alleviated the morphological changes in BV2 cells induced by AβO. ISL treatment further protected N2a cells from the toxic medium of AβO-stimulated BV2 cells. ISL activated nuclear factor erythroid-2 related factor 2 (Nrf2) signaling and suppressed nuclear factor-κB (NF-κB) signaling in BV2 cells.

**Conclusion:**

ISL suppresses AβO-induced inflammation and oxidative stress in BV2 cells *via* the regulation of Nrf2/NF-κB signaling. Therefore, ISL indirectly protects neurons from the damage of toxic conditioned media.

## Introduction

Alzheimer’s disease (AD), a chronic neurodegenerative disorder and the leading cause of dementia, leads to severe cognitive impairment (Holtzman et al., 2011). Senile plaques, which are formed by the deposition of amyloid-β (Aβ), are considered to be the most characteristic pathological changes in Alzheimer’s disease (Selkoe and Hardy, 2016). Extensive evidence has shown that amyloid-β oligomers (AβO), a toxic form of Aβ, promote a series of pathological changes in AD, including synaptic dysfunction, neuroinflammation, mitochondrial dysfunction, and neuronal death (Hu et al., 2017; Caruso et al., 2019). To date, no approved disease-modifying therapies effectively prevent or delay the progression of AD, and almost all clinical trials have failed (Mangialasche et al., 2010; Mehta et al., 2017).

In the past decades, strong evidence has emerged that both neuroinflammation and oxidative stress also contribute to the development of AD (Heneka et al., 2015; Calsolaro and Edison, 2016; Butterfield and Boyd-Kimball, 2020). In fact, several anti-inflammatory drugs or antioxidants have been shown to alleviate the pathological changes and cognitive disorder caused by AD, both *in vivo* and *in vitro* (Teixeira et al., 2013; Calsolaro and Edison, 2016). Neuroinflammation and oxidative stress are typically caused by AβO, and in turn, promote the generation of Aβ (Cai et al., 2014; Cheignon et al., 2018). Importantly, microglia, as the key immune cells in brain, play a vital role in these processes. That is, microglia surround amyloid plaques and respond to Aβ with a pro-inflammatory phenotype (Hansen et al., 2018; Simpson and Oliver, 2020). This microglial phenotype is characterized by excessive cytokine expression, including tumor necrosis factor-α (TNF-α), interleukin-6 (IL-6), and interleukin-1β (IL-1β), resulting in neurotoxicity (Hansen et al., 2018; Simpson and Oliver, 2020). Nuclear factor-κB (NF-κB) is a key transcription factor that upregulates the expression of IL-1β, IL-6, and TNT-α in microglia (Hayden and Ghosh, 2008).

Nitric oxide (NO) is an important gaseous molecule in the nervous system and generated in microglia by the inducible isoform of nitric oxide synthase (iNOS) (Heneka et al., 2001). NO performs numerous functions including the regulation of inflammation and oxidative stress in microglia/macrophage (Caruso et al., 2017; Fresta et al., 2020). In fact, under inflammatory conditions, microglia/macrophage will produce excess NO, accompanied by a high level of reactive oxygen species (ROS). NO can react with ROS (such as superoxide) to generate reactive nitrogen species (RNS). The increased formation of RNS and ROS triggers oxidative/nitrative stress, which is often the pathological characteristic of neurodegenerative disorders. The nuclear factor erythroid-2 related factor 2 (Nrf2) as well as its downstream genes play an important role in the detoxification from ROS and RNS (Crosswhite and Sun, 2010; Caruso et al., 2017; Fresta et al., 2020). Moreover, the Nrf2 pathway also limits inflammation in microglia (Moi et al., 1994; Simpson and Oliver, 2020).

Isoliquiritigenin (ISL), is extracted from the roots of Glycyrrhiza uralensis. It has multiple biological activities, including antioxidative anti-inflammatory properties, as well as possessing antibacterial and anti-diabetic activities, with good ability to penetrate the blood-brain barrier (Ramalingam et al., 2018). Importantly, a previous animal study found that ISL protects against cognitive impairment and neuronal injury induced by injection of lipopolysaccharides (LPS) (Zhu et al., 2019). Link et al. (2015) reported that ISL could effectively inhibit Aβ1-42 aggregation in Caenorhabditis elegans models (Link et al., 2015). Furthermore, Lee et al. (2012) found that ISL protect cortical neurons from neurotoxicity induced by Aβ25-35, and significantly reduced the cellular Ca2 + concentration and ROS levels (Lee et al., 2012). Moreover, it was demonstrated that ISL could activate the Nrf2 responsive antioxidant pathway and inhibit the NF-κB pathway in chronic obstructive pulmonary disease and intracerebral hemorrhage models (Zeng et al., 2017; Yu et al., 2018).

To date, the effects of ISL on Nrf2 signaling and NF-κB signaling in cellular models of AD have not been evaluated. To that end, we stimulated murine microglial cells (BV2) with AβO to build cellular models. We hypothesized that ISL would ameliorate Aβ-induced inflammation and oxidative stress of BV-2 cells by up-regulating Nrf2 signaling and down-regulating NF-κB signaling.

## Materials and Methods

### AβO Preparation and Cell Culture

AβO powder was obtained from China Peptides (Shanghai, China). 1 mg of Aβ42 oligomer powder was dissolved in dimethyl sulfoxide (DMSO) (Sigma-Aldrich, Saint Louis, United States) and then further diluted with Dulbecco’ s modified eagle medium (DMEM) (Gibco, NY, United States) to a final concentration of 5 μM. Based on the previous findings of our team (Chen and Jia, 2020; Zhang et al., 2020), 5 μM AβO was chosen as the intervention concentration in the subsequent experiments. BV2 cells and mouse neuroblastoma (N2a) cells were supplied by the National Infrastructure of Cell Line Resource (Beijing, China). Cell lines were maintained at 37°C and 5% CO2 and cultured with the complete medium, composed of DMEM, 10% fetal bovine serum (Gibco, NY, United States), and 1% penicillin-streptomycin (Sigma-Aldrich, Saint Louis, United States).

### Cell Viability Assays

3-(4,5-dimethyl-2-thiazolyl)-2, 5-diphenyl-2-H- tetrazolium bromide (MTT) (Sigma-Aldrich, Saint Louis, United States) and lactate dehydrogenase (LDH) cytotoxicity assays (Beyotime Biotech, Shanghai, China) were used to assess cell viability after treatment with ISL (Pufei De Biotech, Chengdu, China) or conditioned medium. Briefly, BV2 cells were seeded (5 × 10^3^ cells per well) into 96-well culture dishes (Corning, Beijing, China) and cultured overnight. For MTT assays, we stimulated BV2 cells with 1, 5, 10, and 20 μM ISL for 24 h. Next, we incubated cells with 10 μL MTT (5 mg/ml) at 37°C for 4 h and then replaced the supernatant with 100 μL DMSO. We measured the absorbance at 570 nm using a microplate reader (Thermo Fisher, Vantaa, Finland). To collect the conditioned medium, pre-treating BV2 cells with ISL for 2 h prior to co-culturing with 5 μM AβO for 6 h was required. Then, the medium was discarded and cells were cultured with fresh medium without AβO or ISL for another 12 h to collect the conditioned medium. N2a cells were seeded in 96-well culture dishes (1 × 10^4^ cells per well) and cultured with prepared conditioned medium for 24 h to perform the MTT assay. For the LDH cytotoxicity assay, N2a cells seeded in 96-well culture dishes were cultured with conditioned medium for 24 h and then the medium was collected. The remaining cells were lysed with Triton X-100 (Solarbio Biotech, Beijing, China). The LDH contents of the medium and lysed cells were considered as released LDH and intracellular LDH, respectively, and were measured according to the manufacturer’s instructions.

### Quantitative Real-Time Polymerase Chain Reaction

BV2 cells were seeded (4 × 10^5^ cells per well) into 6-well culture dishes (Corning, Beijing, China) and cultured overnight. Then the cells were pre-treated with ISL for 2 h and co-cultured with 5 μM AβO for another 6 h. Next, we extracted the total RNA from BV2 cells, which was then reverse-transcribed into cDNA using a commercial kit (Takara Biotech, Beijing, China). We used SYBR Green PCR Master Mix (Takara Biotech, Beijing, China) to perform real-time polymerase chain reaction (qRT-PCR) on the Step One Plus Real-time PCR System (Applied Biosystems, Foster City, United States). All steps were performed according to the manufacturer’s instructions. Table 1 shows the oligonucleotide primer sequences used in our study. Relative mRNA levels were normalized by glyceraldehyde 3-phosphate dehydrogenase (GAPDH) within the same samples.

**TABLE 1:** Sequences of oligonucleotide primers.

IL-1β	forward primer	5′-TTTCCTCCTTGCCTCTGATGGG-3′
	reverse primer	5′-CCACACGTTGACAGCTAGGTTC-3′
TNF-α	forward primer	5′-GTGGTCAGGTTGCCTCTGTCTC-3′
	reverse primer	5′-TGGCTCTGTGAGGAAGGCTGTG-3′
IL-6	forward primer	5′-CTTGGGACTGATGCTGGTGACA-3′
	reverse primer	5′-GCCTCCGACTTGTGAAGTGGTA-3′
iNOS	forward primer	5′-GGACGAGACGGATAGGCAGAGA-3′
	reverse primer	5′-TCTTCAAGCACCTCCAGGAACG-3′
COX-2	forward primer	5′-AATGCTGGTGTGGAAGGTGGTG-3′
	reverse primer	5′-GCTCTAGGCTTTGCTGGCTACC-3′
Nrf2	forward primer	5′-AGCACAGCCAGCACATTCTCC-3′
	reverse primer	5′-ACCAGGACTCACGGGAACTTCT-3′
HO-1	forward primer	5′-GACCGCCTTCCTGCTCAACATT-3′
	reverse primer	5′-CCTCTGACGAAGTGACGCCATC-3′
NQO1	forward primer	5′-GCCATGTACGACAACGGTCCTT-3′
	reverse primer	5′-CGCAGGATGCCACTCTGAATCG-3′
GAPDH	forward primer	5′-GAAGGGCATCTTGGGCTACAC-3′
	reverse primer	5′-GTTGTCATTGAGAGCAATGCCA-3′

*IL-1β, interleukin-1β; TNF-α, tumor necrosis factor-α; IL-6, interleukin-6; iNOS, inducible nitric oxide synthase; COX-2, cyclooxygenase-2; Nrf2, nuclear factor erythroid-2 related factor 2; HO-1, heme oxygenase-1; NQO1, NAD(P)H: quinone oxidoreductase-1; GADPH, glyceraldehyde 3-phosphate dehydrogenase.*

### Nitric Oxide (NO) Assay and Enzyme-Linked Immunosorbent Assays

The level of NO was assessed indirectly by measuring its end-products, nitrate and nitrite. BV2 cells were seeded into 6-well culture dishes (4 × 10^5^ cells per well) and cultured overnight. Then, BV2 cells were pre-treated with ISL for 2 h, then co-treated with 5 μM AβO for 24 h. After treatment, the supernatant was collected and measured according to the instructions of the NO detection kit (Nanjing Jiancheng Institute of Bioengineering, Nanjing, China).

BV2 cells were plated in 6-well culture plates (4 × 10^5^ cells per well) in the culture medium. Then the cells were pre-treated with ISL for 2 h and co-treated with 5 μM AβO for 24 h. Next, we used the commercial enzyme-linked immunosorbent assays (ELISA) kits (CUSABIO Technology, Wuhan, China) to measure the release of IL-1β, IL-6, and TNF-α in the cell supernatant according to the manufacturer’s protocol.

### Cell Transfection

Small interfering RNA (siRNA) (Ribobio, Guangzhou, China) was used to transfect BV-2 cells in order to knockdown the expression of Nrf2. The target sequence of the Nrf2 siRNA was 5′-CGACAGAAACCTCCATCTA-3′. siRNA was mixed with transfection reagent (Ribobio, Guangzhou, China) and applied to BV2 cells at 50 nM for 24 h according to the manufacturer’s protocol.

### Western Blot

BV2 cells were cultured in 6-well plates (2 × 10^5^ cells per well) and transfected by Nrf2 siRNA. Then, BV2 cells were pre-treated with ISL for 2 h, and then co-treated with 5 μM AβO for 24 h to detect the expression of Nrf2, heme oxygenase-1 (HO-1), and NAD(P)H: quinone oxidoreductase-1 (NQO1) or 1 h to detect the expression of NF-κB. Next, we used radioimmunoprecipitation assay buffer (Applygen Biotechnology, Beijing, China) to extract the total protein or used the commercial kit (Applygen Biotechnology, Beijing, China) to extract the nuclear and cytosolic proteins from BV-2 cells. The protein concentration was then measured using a bicinchoninic acid protein assay kit (Applygen Biotechnology, Beijing, China). The protein samples were separated by 10% sodium dodecyl sulfate polyacrylamide gel electrophoresis (Applygen Biotechnology, Beijing, China) and transferred to polyvinylidene difluoride membranes (Millipore, Billerica, MA, United States). The membranes were blocked using 5% non-fat dry milk (Solarbio Biotech, Beijing, China) for 1 h at room temperature and then incubated with primary antibodies against Nrf2 (1:1,000, Abcam, United States), HO-1 (1:1,000, Abcam, United States), NQO1 (1:1,000, Abcam, United States), NF-κB p65 (1:1,000, Abcam, United States), β-actin (1:1,000, ZSGB Biotech, Beijing, China), and lamin B1 (1:1,000, Proteintech Biotechnology, Wuhan, China) at 4°C overnight. The membranes were incubated with secondary antibody (1:3,000, ZSGB Biotech, Beijing, China) at room temperature for 1 h. The blots were observed using enhanced chemiluminescent reagent (R&D Systems) and analyzed using Image J software (NIH, Bethesda, MD, United States).

### Immunofluorescence Staining and Morphological Characterization

BV2 cells were seeded (1 × 10^4^ per well) into 48-well plates (Corning, Beijing, China). The cells were pre-treated with ISL for 2 h, and then co-treated with 5 μM AβO for 24 h. After washing with phosphate buffer solution (PBS) (Hyclone, Logan, United States) three times, we fixed the BV2 cells with 4% paraformaldehyde (Sigma-Aldrich, Saint Louis, United States) for 15 min at room temperature and then washed with PBS again. Next, we used 0.3% Triton X-100 to permeabilize the cells and the cells were blocked with 10% goat serum (Solarbio Biotech, Beijing, China). Then, the cells were incubated with primary antibodies against NF-κB p65 (1:100, Abcam, United States) or Nrf2 (1:200, Abcam, United States) overnight at 4°C and incubated with Alexa Fluor 488 goat anti-mouse IgG (1:200, ZSGB Biotechnology, Beijing, China) for 1 h at room temperature. The nuclei were stained with 4′,6-diamidino-2-phenylindole (DAPI) (Beyotime Biotechnology, Shanghai, China). Finally, we mounted the cells onto slides with Antifade Mounting Medium (Beyotime Biotechnology, Shanghai, China), and observed and collected images using a fluorescent microscope. For observation of morphological characterization, BV2 cells seeded in 48-well plates (1.5 × 10^4^ per well) were pre-treated with ISL for 2 h, and then co-treated with 5 μM AβO for 6 h. Then, BV2 cells were incubated with primary antibodies against ionized calcium binding adapter molecule 1 (Iba1) (1:100, Proteintech Biotechnology, Wuhan, China) overnight at 4°C and incubated with Alexa Fluor 488 goat anti-mouse IgG (1:200, ZSGB Biotechnology, Beijing, China) for 1 h at room temperature. Next, the cells were incubated with ActinRed (1:50, Keygen Biotechnology, Nanjing, China) for 1 h at room temperature to mark the cytoskeleton and incubated with DAPI for 15 min to mark the nucleus. We used Image J software (NIH, Bethesda, MD, United States) to analyze the morphology of BV2 cells.

### Statistical Analysis

All data are presented as mean ± standard deviation (SD). GraphPad Prism version 7.0 (GraphPad Prism Software Inc., San Diego, CA, United States) was used to perform the statistical analysis. One-way ANOVA with Tukey’s *post hoc* test was used to detect group differences. p values < 0.05 were considered as statistically significant.

## Results

### ISL Attenuates AβO Induced Inflammation in BV2 Cells

**FIGURE 1:**
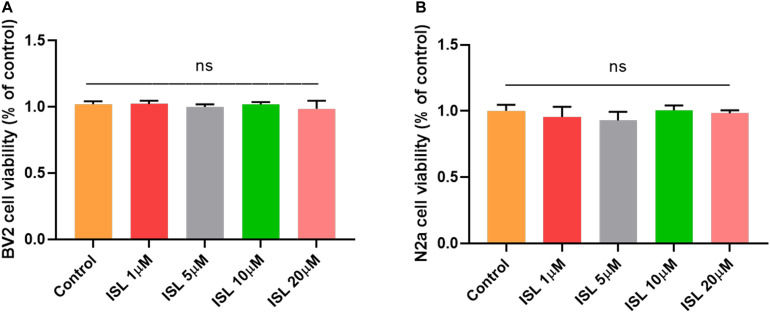
The effects of Isoliquiritigenin on cell viability. **(A)** Cell viability of BV2 cells treated with ISL for 24 h was assessed by MTT assays. **(B)** Cell viability of N2a cells treated with ISL for 24 h was assessed by MTT assays. ns, not significant (*p* > 0.05). *n* = 6 per group.

**FIGURE 2:**
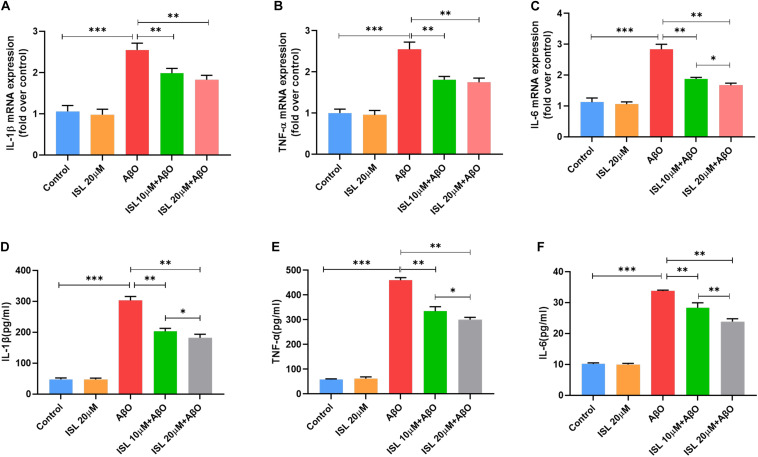
ISL inhibited the production of pro-inflammatory cytokines in BV2 cells induced by AβO. **(A–C)** BV2 cells were pre-treated with ISL for 2 h and then co-treated with 5 μM AβO for 6 h. The mRNA levels of IL-1β, IL-6, and TNF-α in BV2 cells treated with AβO in the presence or absence of ISL were analyzed by qRT-PCR. **(D–F)** BV2 cells were pre-treated with ISL for 2 h and then co-treated with 5 μM AβO for 24 h. The production of TNF-α, IL-6, and IL-1β in BV2 cells treated with AβO in the presence or absence of ISL were determined by ELISA. *p < 0.05, **p < 0.01, ***p < 0.001. *n* = 6 per group.

As shown in Figures 1A,B, none of the concentrations of ISL (0–20 μM) produced cytotoxicity in BV2 or N2a cells assessed by the MTT assay. As shown in Figures 2A–C, the mRNA levels of IL-1β, TNF-α, and IL-6 in BV-2 cells significantly increased after AβO stimulation, compared with the non-treated group (all *p* < 0.001). Notably, pre-treating BV2 cells with 10 μM and 20 μM ISL decreased the mRNA levels of above-mentioned cytokines (vs. AβO treated group, Figures 2A–C, all *p* < 0.01). There is a significant difference between ISL 10 μM pre-treated cells and the ISL 20 μM pre-treated cells in the mRNA expression of IL-6. When BV2 cells were pre-treated with 20 μM ISL, the reduction of mRNA levels of IL-1β, TNF-α, and IL-6 reached 28.3%, 31.4%, and 41.1%, respectively (Figures 2A–C, all *p* < 0.01). No statistically significant differences were observed between the ISL treated group and the control group.

We further used ELISA to examine the release of IL-1β, IL-6, and TNF-α in groups with different treatments. As illustrated in Figures 2D–F, the levels of IL-1β, IL-6, and TNF-α increased significantly after Aβ stimulation compared to those in the non-treated cells (all *p* < 0.001). Pre-treating BV2 cells with 10 and 20 μM ISL decreased the levels of IL-1β, IL-6, and TNF-α (vs. AβO treated group, Figures 2D–F, all *p* < 0.01) in a dose-dependent manner. 20 μM of ISL pre-treatment attenuated Aβ-induced secretion of these inflammatory cytokines (Figures 2D–F) by approximately 39.9% (for IL-1β, *p* < 0.01), 29.3% (for IL-6, *p* < 0.01), and 34.7% (for TNF-α, *p* < 0.01), as compared with AβO treated group. No statistically significant differences were observed between the ISL treated group and the control group.

**FIGURE 3:**
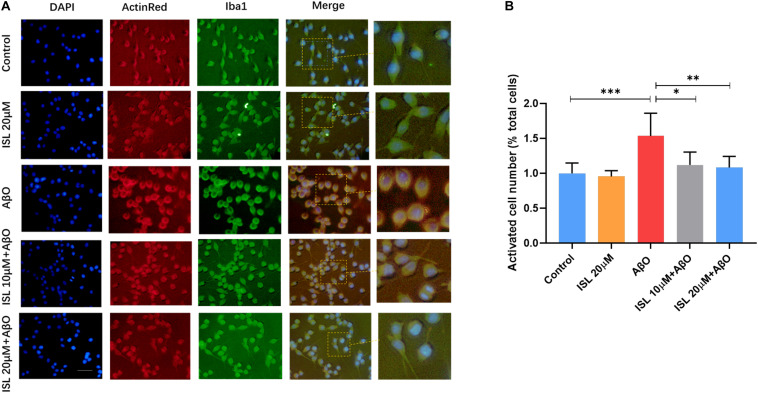
ISL suppressed the morphological changes of BV2 cells exposed to AβO. BV2 cells were pre-treated with 20 μM ISL for 2 h and then co-treated with 5 μM AβO for 6 h. **(A)** Iba1 and Actin Red was used to assess the morphological changes of BV2 cells. **(B)** ISL treatment suppressed the morphological changes in BV2 cells induced by AβO. Scale bar: 50 μm. *p < 0.05, **p < 0.01, ***p < 0.001.

We also analyzed the morphological alterations of BV2 cells in different groups. As shown in Figure 3A, AβO-stimulated BV2 cells showed larger soma with fewer and shorter branches, characterized as “activated” microglia, compared to those in the non-treated cells (Figures 3A,B, *p* < 0.001). However, ISL 10 or 20 μM pre-treatment significantly reversed the morphological changes, compared with the AβO stimulation group (Figures 3A,B, *p* < 0.05 and *p* < 0.01).

### ISL Regulates the Oxidative Stress in AβO Treated BV2 Cells

**FIGURE 4:**
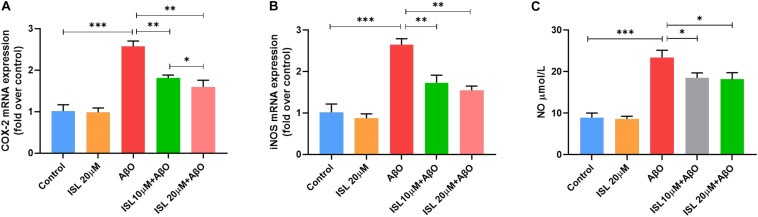
ISL attenuates oxidative stress in BV2 cells. **(A,B)** BV2 cells were pre-treated with ISL for 2 h and then treated with 5 μM AβO for 6 h. The mRNA levels of COX-2 and iNOS were analyzed by qRT-PCR. **(C)** BV2 cells were pre-treated with ISL for 2 h and then treated with 5 μM AβO for 24 h. The production of NO was assessed using the Griess reaction assay. *p < 0.05, **p < 0.01, ***p < 0.001. *n* = 6 per group.

We evaluated the effects of ISL pre-treatment on the mRNA expression of cyclooxygenase-2 (COX-2) and inducible nitric oxide synthase (iNOS) and NO production. AβO treatment in BV2 cells increased mRNA expression of iNOS by 1.6 times (Figure 4A, *p* < 0.001), and COX-2 by 1.5 times (Figure 4B, *p* < 0.001) compared to non-treated cells. However, pre-treating BV2 cells with ISL significantly downregulated the mRNA levels of iNOS and COX-2. The average mRNA expression of iNOS in the ISL pre-treatment group was 34.6% (for 10 μM ISL), and 41.6% (for 20 μM ISL) lower than those in AβO-treated cells (Figure 4A, all *p* < 0.01). Pre-treating cells with ISL reduced the mRNA levels of COX-2 to 29.5% (for 10 μM ISL), and 38.0% (for 20 μM ISL) compared to the group stimulated by AβO alone (Figure 4B, all *p* < 0.01). Additionally, there is a significant difference between ISL 10 μM pre-treated cells and the ISL 20 μM pre-treated cells in the mRNA expression of COX-2 (Figure 4A, *p* < 0.05). Furthermore, stimulating BV2 cells with AβO significantly upregulated the production of NO (Figure 4C, 8.94 ± 1.09 vs. 23.43 ± 1.74, *p* < 0.001), while 10 or 20 μM ISL treatment significantly reduced NO production compared to the AβO-treated group (Figure 4C, both *p* < 0.05). No statistically significant differences were observed between the ISL treated group and the control group.

### ISL Protects N2a Cells Against AβO Induced Neurotoxicity Indirectly

**FIGURE 5:**
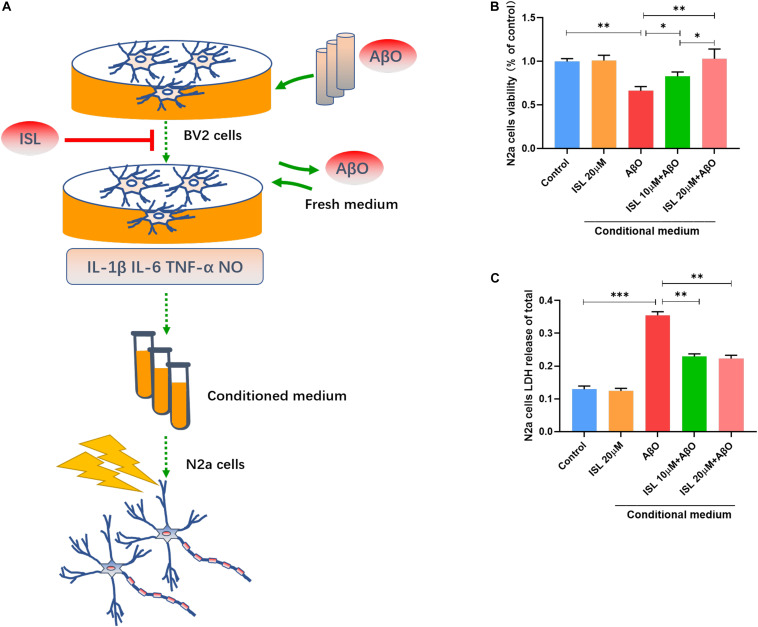
Neuroprotective effects of ISL on N2a cells cultured with conditioned medium from AβO stimulated BV2 cells. **(A)** A diagram of the toxic effect of conditioned media from AβO stimulated BV2 cells on N2a cells with or without the ISL pre-treatment. **(B,C)** N2a cells were treated with conditioned medium for 24 h and cell viability was assessed using MTT and LDH cytotoxicity assays. *p < 0.05; **p < 0.01, ***p < 0.001. *n* = 6 per group.

We verified that ISL treatment could down-regulate the production of inflammatory cytokines and NO in AβO treated cells. We further investigated whether ISL could protect N2a cells from AβO-induced neurotoxicity (Figures 5A–C). Treatment of N2a cells with conditioned medium (Figure 5A), which was extracted from BV2 cells stimulated by AβO, significantly decreased N2a cell viability by 33.9% (vs. control, *p* < 0.01, Figure 5B) and increased LDH release by 1.8 times (vs. control, *p* < 0.001; Figure 5C). However, treatment of N2a cells 10 or 20 μM ISL-pre-treated conditioned medium significantly improved cell viability (vs. AβO-exposed conditioned medium group, *p* < 0.05, and *p* < 0.01, respectively) in a dose-dependent manner and decreased LDH release (vs. AβO-exposed conditioned medium group, all *p* < 0.01). No statistically significant differences were observed between the ISL treated group and the control group.

### ISL Activated the Nrf2 Pathway in AβO Treated BV2 Cells

**FIGURE 6:**
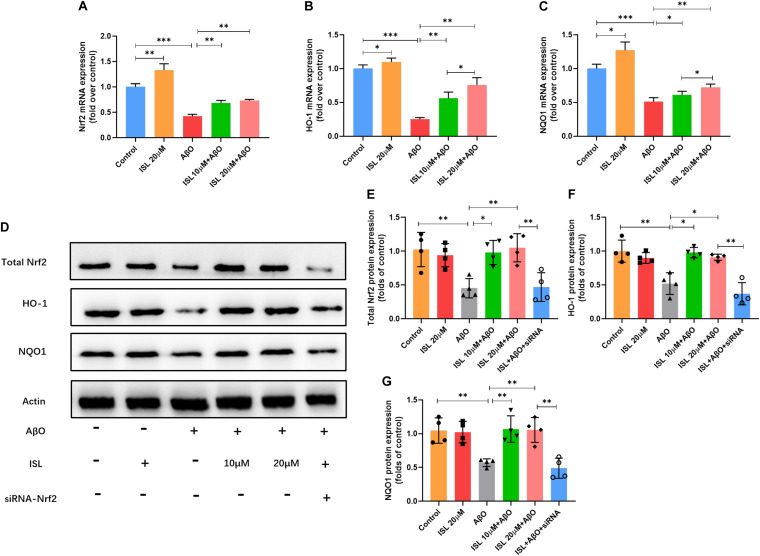
ISL attenuates AβO induced suppression of the Nrf2 pathway. **(A–C)** BV2 cells were pre-treated with ISL for 2 h and then co-treated with 5 μM AβO for 6 h. The mRNA levels of Nrf2, HO-1 and NQO1 were analyzed by qRT-PCR. **(D–G)** BV2 cells, including those transfected by Nrf2 siRNA, were pre-treated with ISL for 2 h and then co-treated with 5 μM AβO for 24 h. Total protein expression of Nrf2, HO-1 and NQO1 were analyzed by Western blot. *p < 0.05, **p < 0.01, ***p < 0.001. *n* = 6 per group for qRT-PCR, *n* = 4 per group for Western blot.

**FIGURE 7:**
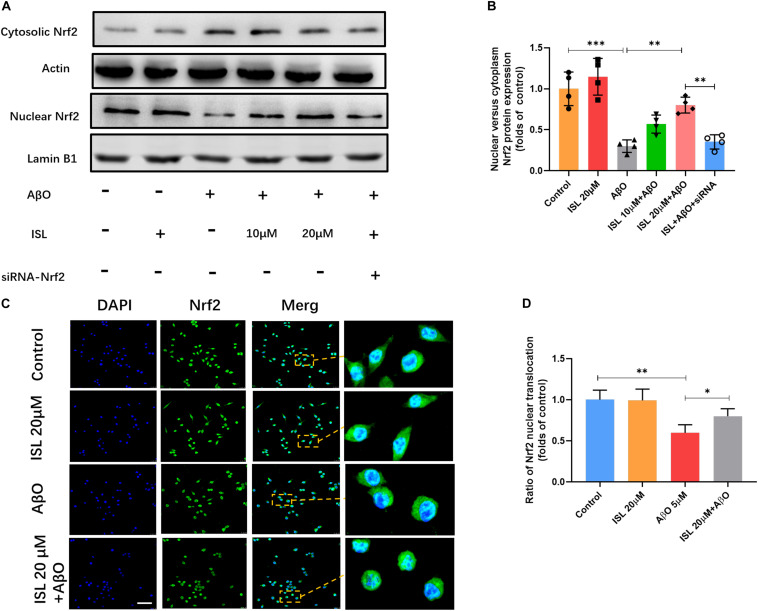
The expression of Nrf2 in nucleus and cytoplasm. BV2 cells, including those transfected by Nrf2 siRNA, were pre-treated with ISL for 2 h and then co-treated with 5 μM AβO for 24 h. **(A,B)** Protein expression of cytosolic and nuclear Nrf2 were analyzed by Western blot. **(C,D)** The translocation of Nrf2 (green) to the nucleus (blue) is shown by confocal microscopic images. Scale bar: 50 μm. *p < 0.05, **p < 0.01, ***p < 0.001. *n* = 4 per group for Western blot and *n* = 6 for immunofluorescence staining.

We next examined whether ISL activated the Nrf2 pathway. As shown in Figures 6A–C, a significantly increased mRNA levels of Nrf2, HO-1 and NQO-1 were observed in the ISL treated group compare to the control group. Our data showed lower mRNA levels of Nrf2, HO-1 and NQO-1 in AβO treated group compared to those in non-treated group (Figures 6A–C, all *p* < 0.001). On the other hand, an increase in mRNA levels in the 10 or 20 μM ISL pre-treated groups was observed compared to those in the AβO treated group (Figures 6A–C, *p* < 0.05 or *p* < 0.01) and the increase in mRNA levels of HO-1 and NQO-1 was in a dose-dependent manner. The results further showed that the AβO-treated BV2 cells had significantly lower total Nrf2 protein expression (Figures 6D,E; *p* < 0.01) and significantly lower nuclear translocation of Nrf2, which is validated by western blot or immunofluorescence staining (vs. control group, Figures 7A–D, *p* < 0.001, and *p* < 0.01, respectively). The HO-1 and NQO-1 expression were also significantly lower in cells treated with AβO (vs. control group, both *p* < 0.01), as shown in Figures 6D,F,G. Pre-treatment with 10 or 20 μM ISL enhance the total protein expression of Nrf2, HO-1, and NQO-1 significantly, compared to those in the group treated with AβO alone (Figures 6D–G, *p* < 0.05 or *p* < 0.01), among which pre-treatment with 20 μM ISL upregulated the protein expression of total Nrf2, HO-1, and NQO-1 by 1.3 times, 0.75 times, and 0.93 times, respectively. Similarly, pre-treatment with 20 μM ISL significantly promoted the nuclear translocation of Nrf2 (vs. AβO treated group, Figures 7A–D, *p* < 0.01 and *p* < 0.05) as indicated by western blot and immunofluorescence staining. Importantly, when cells were cultured with siRNA to knock down Nrf2 expression, the effects of ISL on protein expression of Nrf2, HO-1, and NQO-1 (vs. 20 μM ISL + AβO treated group, all *p* < 0.01, Figures 6D–G) and the nuclear translocation of Nrf2 (vs. 20 μM ISL + AβO treated group, *p* < 0.01, Figures 7A,B) were attenuated. No statistically significant differences were observed between the ISL treated group and the control group, as determined by Western blot and immunofluorescence analysis.

### ISL Suppresses the NF-κB Signaling by Activating the Nrf2 Pathway

**FIGURE 8:**
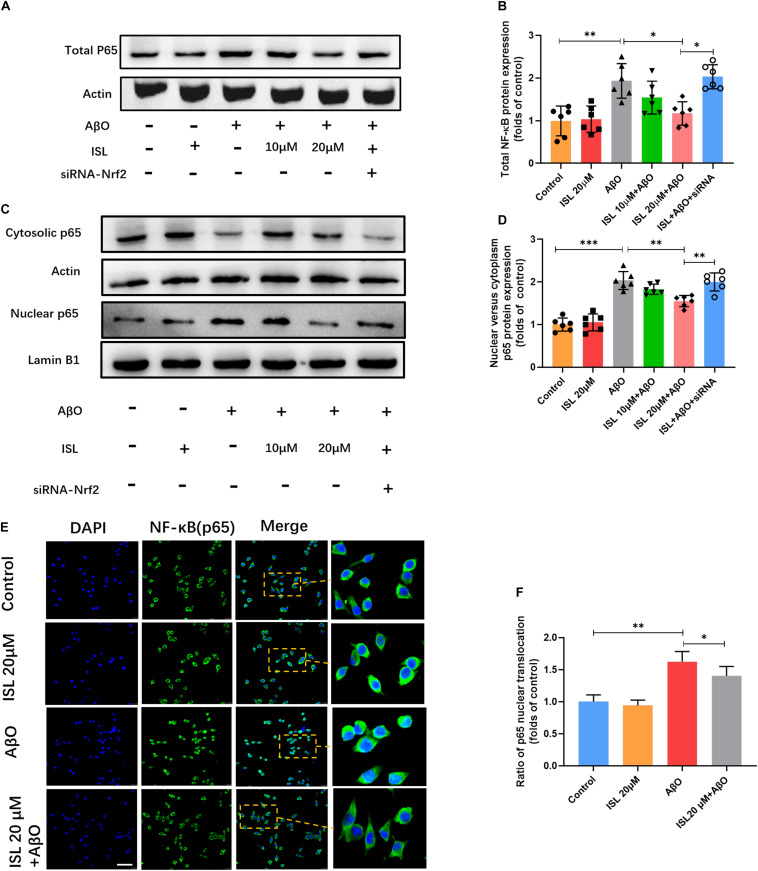
ISL attenuates AβO induced activation of the NF-κB pathway in BV2 cells. **(A–D)** BV2 cells, including those transfected by Nrf2 siRNA, were pre-treated with ISL for 2 h and then co-treated with 5 μM AβO for 1 h. Protein expression of total, cytosolic and nuclear NF-κB were analyzed by Western blot. **(E,F)** BV2 cells, including those transfected by Nrf2 siRNA, were pre-treated with ISL for 2 h and then co-treated with 5 μM AβO for 24 h. The translocation of NF-κBp65 (green) to the nucleus (blue) is shown by confocal microscopic images. Scale bar: 50 μm. *p < 0.05, **p < 0.01, ***p < 0.001. *n* = 6 per group.

To clarify whether ISL can inhibit the NF-κB pathway by regulating the Nrf2 pathway, we analyzed the expression of NF-κBp65 and its translocation into the nucleus. As shown in Figure 8, AβO treatment remarkably increased the expression of NF-κBp65 (Figures 8A,B, *p* < 0.01) and promoted nuclear translocation (Figures 8C–F, *p* < 0.001 and *p* < 0.01) as indicated by western blot and immunofluorescence staining, compared to control groups. However, pre-treatment with 20 μM ISL decreased the expression of NF-κBp65 by 41.9% (Figures 8A,B, *p* < 0.05) and its translocation into the nucleus by 23.7% in western blot (Figures 8C,D, *p* < 0.01) or 13.6% in immunofluorescence staining (Figures 8E,F, *p* < 0.05), compared to the AβO-treated groups. We then assessed the effects of Nrf2 siRNA on the protein expression and nuclear translocation of NF-κBp65. We found that Nrf2 siRNA interference significantly increased the expression of NF-κBp65 and its nuclear translocation (vs. 20 μM ISL + AβO treated group, *p* < 0.05, and *p* < 0.01, respectively, Figures 8A–D), which indicated that ISL suppressed NF-κB signaling by activating the Nrf2 pathway.

## Discussion

Our study revealed that ISL attenuates neuroinflammation and oxidative stress in AβO-treated BV2 cells, including decreasing production of IL-6, IL-1β, TNF-α, and NO. Through these effects, ISL indirectly protects N2a cells against AβO-induced neurotoxicity. Next, we found that ISL effectively activated the Nrf2 pathway and inhibited the NF-κB pathway, thereby suppressing the production of inflammatory cytokines and NO in microglia stimulated by AβO.

Previous studies have shown that microglia can be stimulated by fibrillar Aβ to induce the production of TNFα, IL-1β, and IL-6 (Heneka et al., 2014). This result was verified in our cellular models as stimulation with AβO was sufficient to activate microglial BV2 cells and increase the production of TNF-α, IL-1β, and IL-6 (see Figure 2). Microglial cells are known to respond to these inflammatory cytokines, thereby impairing their ability to clear amyloid-β, resulting in Aβ deposition (Domingues et al., 2017; Hansen et al., 2018). In fact, excessive production of IL-1β has also been demonstrated to disrupt dendritic spine formation and interfere with memory consolidation (Tong et al., 2012). Moreover, IL-1β and TNF-α derived from microglia have been shown to reduce neurogenesis (Voloboueva and Giffard, 2011; Zou and Crews, 2012)and induce apoptosis in hippocampal neural precursor cells (Cacci et al., 2005; Guadagno et al., 2015). Thus, early immunosuppression of these cytokines might lead to reduce Aβ pathology and neurotoxicity (Bhaskar et al., 2010). In our study (see Figures 2, 3), ISL pre-treatment decreased the mRNA expression and secretion of these cytokines, as well reversed the morphological changes, indicating that ISL might play a neuroprotective role against neuroinflammation.

iNOS has been shown to be expressed in microglial cells in response to pro-inflammatory cytokines (Heneka et al., 2001). NO, primarily produced by iNOS, is suggested to play a role in axonal and synaptic damage, mitochondrial injury, and neuronal apoptosis (Beal, 2000; Nakamura and Lipton, 2009). Importantly, the NO reaction product can nitrate the tyrosine residue of the Aβ peptide (Kummer et al., 2011). Nitrated Aβ increases the tendency of aggregation and the ability to suppress synaptic plasticity, compared with non-nitrated Aβ (Kummer et al., 2011). COX-2 is an enzyme subunit that mediates the production of ROS. It is also expressed in microglia and is activated by pro-inflammatory stimulation (Simpson and Oliver, 2020). Elevation of COX-2 activity is known to be related to the pathological changes in Aβ and tau, neuronal loss, neuroinflammation, and oxidative stress in AD (Guan and Wang, 2019). In this study (see Figure 4), ISL pre-treatment reduced the elevation of iNOS and COX-2 expression and NO production in AβO-stimulated BV2 cells. Moreover, culturing N2a cells with ISL pre-treated conditioned medium significantly improved cell viability, which might be due to the reduction of inflammatory factors and NO released from activated BV2 cells (see Figure 5).

We have demonstrated the anti-inflammatory activity of ISL in cell models exposed to AβO. In terms of the underlying molecular mechanisms, we further demonstrated that ISL decreased expression of total NF-κB p65 and suppressed NF-κB p65 translocation from the cytosol into the nucleus in BV2 cells exposed to AβO (see Figure 8). These major findings indicate that ISL might suppress inflammation in microglia by inhibiting NF-κB activation. NF-κB is a key transcription factor that regulates the inflammatory response and consists of various subunits including RelA (p65) (Hayden and Ghosh, 2008). Under extracellular signals, NF-κB dissociates from IκB proteins and then binds to various promoters in the nucleus to regulate the expression of TNF-α, IL-6, IL-1β, iNOS, and COX-2 (Hayden and Ghosh, 2008; Cai et al., 2014). Stimulation, including by IL-1β and TNF-α, can then trigger this pathway forming a vicious circle (Shi et al., 2016). Moreover, previous studies have indicated that upregulation of NF-κB activity also aggravates the production of Aβ, thereby participating in the development of AD (Valerio et al., 2006; Chami et al., 2015). Therefore, suppressing NF-κB signaling in AD may prevent the amplification of the inflammatory cascade and neurodegeneration.

Nrf2 exerted a crucial effect on maintaining the balance between oxidative stress and oxidation resistance (Moi et al., 1994; Itoh et al., 1997; Jung and Kwak, 2010; Sharma et al., 2020). In cytoplasm, Nrf2 binds with kelch like ECH related protein 1 (Keap1) to inhibit its activation. After exposure to ROS or other stimulations, the Keap1-Nrf2 protein-protein interaction is interrupted. Subsequently, the isolated Nrf2 protein is transferred into the nucleus and induce the transcription of NQO1 and HO-1 (Itoh et al., 1997; Jung and Kwak, 2010). Although evidence of increased ROS has been identified in AD, several studies demonstrate that this endogenous protective pathway is down-regulated in AD models and patients (Ramsey et al., 2007; Tian et al., 2019; Sharma et al., 2020), which may be explained by different stage of AD (Fao et al., 2019). In this study (see Figures 6, 7) we found that the mRNA and protein levels of Nrf2, HO-1, and NQO1 and nuclear translocation of Nrf2 significantly decreased under AβO stimulation, which was consistent with previous studies (Ali et al., 2018; Chiang et al., 2018; Li et al., 2020). However, ISL pre-treatment notably promoted the expression of Nrf2, HO-1, and NQO1 at both the transcription and translation levels as well as promoted the nuclear translocation of Nrf2 (see Figures 6, 7). Furthermore, activating Nrf2 pathway has been shown to inhibit oxidative stress, reduce Aβ, and improve the cognitive function of mice (Kanninen et al., 2009; Tian et al., 2019). In contrast, blocking this pathway increases oxidative damage and deteriorates cognitive function (Branca et al., 2017; Tian et al., 2019). Taken together, these results demonstrate and emphasize the indispensable role of Nrf2 pathway on regulating oxidative stress in AD.

Moreover, recent studies have established the existence of crosstalk between Nrf2 and NF-κB signaling under several pathophysiological conditions (Wardyn et al., 2015). In microglia, deletion of Nrf2 is associated with augmentation of inflammation triggered by cytokines in response to LPS, while intracranial injection of Nrf2 activators decreases the levels of pro-inflammatory mediators and exerts a neuroprotective effect (Innamorato et al., 2008; Wardyn et al., 2015). Further studies have shown that knockout of Nrf2 increases the activity of inhibitor kappa B kinase β and enhances the phosphorylation of IκBα (Thimmulappa et al., 2006). In this study (see Figure 8), we found that Nrf2 siRNA partially suppressed the inhibitory effect of ISL on the NF-κB signaling induced by AβO in terms of expression and nuclear translocation of NF-κB (p65), indicating that ISL treatment suppresses NF-κB pathway by activating the Nrf2 signaling pathway. From the current and previous results, the strategy of restoring Nrf2 activity (thus inhibiting NF-κB activation) represents a feasible treatment for AD and other neurodegenerative diseases. Therefore, the development of Nrf2 chemical activators, including ISL, as antioxidant inflammation modulators should be considered. However, more experiments need to be carried out to evaluate the actual values on AD.

Several studies have reported that ISL has neuroprotective activities in cellular models of neurodegenerative diseases (Ramalingam et al., 2018). For example, ISL protects neuronal cells from neurotoxicity of 6-hydroxydopamine by alleviating the generation of ROS and reactive nitrogen species (RNS) and improving the mitochondrial membrane potential (Hwang and Chun, 2012). ISL protects cortical neurons from neurotoxicity induced by Aβ (25–35) (Lee et al., 2012). To the best of our knowledge, our study is the first to show that ISL exerts an indirect neuroprotective effect through anti-inflammatory and antioxidant properties in a cellular model of AD by AβO stimulation. Moreover, this is the first study to reveal the role of ISL on regulating the Nrf2 and NF-κB pathways in this cellular model, and further explore the internal connection between inflammation and oxidative stress in terms of Nrf2 and NF-κB pathways. More importantly, our results provide evidence for Nrf2/NF-κB signaling as a therapeutic target and Nrf2 activator as a potential treatment for AD. However, as our study was focused on cellular models, the neuroprotective functions and molecular mechanisms have not been verified *in vivo*. Therefore, studies should be carried out to evaluate the protective effect and explore the molecular mechanisms underlying ISL in animal models of AD.

In conclusion, our study demonstrated that ISL can suppress the increase of inflammatory cytokines and NO in AβO-treated BV2 cells by upregulating the Nrf2 pathway and downregulating the NF-κB pathway. Considering that ISL protects N2a cells against AβO-induced neurotoxicity through these effects, ISL may represent a novel therapeutic approach for AD.

## Data Availability Statement

The original contributions presented in the study are included in the article/supplementary material, further inquiries can be directed to the corresponding author/s.

## Author Contributions

YF designed and performed the experiments, analyzed the data, and wrote the manuscript. JJ supervised the research, revised the manuscript, and obtained the funding. Both authors contributed to the article and approved the submitted version.

## Conflict of Interest

The authors declare that the research was conducted in the absence of any commercial or financial relationships that could be construed as a potential conflict of interest.
